# Information-Length Scaling in a Generalized One-Dimensional Lloyd’s Model

**DOI:** 10.3390/e20040300

**Published:** 2018-04-20

**Authors:** J. A. Méndez-Bermúdez, R. Aguilar-Sánchez

**Affiliations:** 1 Instituto de Física, Benemérita Universidad Autónoma de Puebla, Puebla 72570, Mexico; 2 Facultad de Ciencias Químicas, Benemérita Universidad Autónoma de Puebla, Puebla 72570, Mexico

**Keywords:** Lloyd model, scaling laws, information length, one-dimensional disordered systems

## Abstract

We perform a detailed numerical study of the localization properties of the eigenfunctions of one-dimensional (1D) tight-binding wires with on-site disorder characterized by long-tailed distributions: For large ϵ, $P\left(\epsilon \right)∼1/\epsilon^{1+\alpha }$ with α∈(0,2]; where ϵ are the on-site random energies. Our model serves as a generalization of 1D Lloyd’s model, which corresponds to α=1. In particular, we demonstrate that the information length β of the eigenfunctions follows the scaling law β=γx/(1+γx), with x=ξ/L and γ≡γ(α). Here, ξ is the eigenfunction localization length (that we extract from the scaling of Landauer’s conductance) and *L* is the wire length. We also report that for α=2 the properties of the 1D Anderson model are effectively reproduced.

## 1. Introduction

There is a class of disordered systems characterized by random variables {ϵ} whose density distribution function exhibits a slow decaying tail:

(1) $$P\left(\epsilon \right)∼\frac{1}{{\left|\epsilon \right|}^{1+\alpha }}\;,$$

for large |ϵ|, with 0<α<2. The study of this class of disordered systems dates back to Lloyd [1], who studied spectral properties of a three-dimensional (3D) lattice described by a 3D tight-binding Hamiltonian with Cauchy-distributed on-site potentials (which corresponds to the particular value α=1 in Equation (1)). Since then, a considerable number of works have been devoted to the study of spectral, eigenfunction, and transport properties of Lloyd’s model in its original 3D setup [2,3,4,5,6,7,8,9,10,11,12] and in lower dimensional versions [11,12,13,14,15,16,17,18,19,20,21,22,23,24,25,26,27,28,29,30]. Consequently, disorder characterized by Equation (1) is commonly known as Lévy-type disorder. In addition, the recent experimental realizations of the so-called Lévy glasses [31] as well as Lévy waveguides [32,33] has refreshed the interest in the study of systems characterized by Lévy-type disorder; see some examples in Refs. [34,35,36,37,38,39,40,41,42,43,44,45,46,47,48,49,50].

It is important to point out that one-dimensional (1D) tight-binding wires with power-law distributed random on-site potentials, characterized by power-laws different from α=1 (which corresponds to the 1D Lloyd’s model), have been scarcely studied; for prominent exceptions see Refs. [26,27]. Thus, in this paper, we perform a detailed numerical study of the localization properties of the eigenfunctions of disordered wires defined as a generalization of the 1D Lloyd’s model as follows. We shall study 1D wires described by the Hamiltonian

(2) $$H=\sum_{n=1}^{L}\left[\epsilon_{n}|n\rangle \langle n|-\nu_{n,n+1}|n\rangle \langle n+1|-\nu_{n,n-1}|n\rangle \langle n-1|\right];$$

where *L* is the length of the wire given as the total number of sites *n*, $\epsilon_{n}$ are random on-site potentials, and $\nu_{n,m}$ are the hopping integrals between nearest neighbours. In particular, we set $\nu_{n,m}=\nu =1$ and consider the on-site potentials $\epsilon_{n}$ following the distribution of Equation (1) with 0<α≤2. We note that when α=1 we recover the original 1D Lloyd’s model.

Of particular interest is the comparison between the 1D Anderson model [51] and our generalization of 1D Lloyd’s model, since the former represents the most prominent model of disordered wires [52]. Indeed, the 1D Anderson model is also described by the tight-binding Hamiltonian of Equation (2). However, while for the standard 1D Anderson model (with white-noise on-site disorder $\langle \epsilon_{n}\epsilon_{m}\rangle =\sigma^{2}\delta_{nm}$ and $\langle \epsilon_{n}\rangle =0$) the on-site potentials are characterized by a finite variance $\sigma^{2}=\langle \epsilon_{n}^{2}\rangle$ (in most cases the corresponding probability distribution function P(ϵ) is chosen as a box or a Gaussian distribution), in the generalized 1D Lloyd’s model the second moment of the random on-site energies $\epsilon_{n}$ diverges for 0<α<2, and if 0<α<1 also the first moment diverges. Moreover, when α=2 the properties of the generalized 1D Lloyd’s model are expected to be similar to those of the 1D Anderson model, since the variance of Equation (1) becomes finite.

It is relevant to recall that the eigenstates Ψ of the *infinite* 1D Anderson model are exponentially localized around a site position $n_{0}$ [52]:

(3) $$|\Psi_{n}|∼\exp\left(-\frac{|n-n_{0}|}{\xi }\right)\;;$$

where ξ is the eigenfunction localization length. Moreover, for weak disorder ($\sigma^{2}\ll 1$), the only relevant parameter for describing the statistical properties of the transmission of the *finite* 1D Anderson model is the ratio L/ξ [53], a fact known as single parameter scaling. The above exponential localization of eigenfunctions strongly affects the scattering and transport properties of the corresponding open wire. In particular, the transmission or dimensionless conductance *G*, that within a scattering approach to the electronic transport is given as [54,55,56,57]

(4) $$G={\left|t\right|}^{2}$$

(where *t* is the transmission amplitude of the 1D wire), becomes exponentially small [58]:

(5) $$\langle -\ln G\rangle =\frac{2}{x}\;$$

with

(6) $$x=\frac{\xi }{L}\;.$$

Thus, relation (5) can be used to obtain the localization length ξ from the transmission of the disordered wire. Remarkably, it has been shown that Equation (5) is also valid for the generalized 1D Lloyd’s model [26,27] implying the single parameter scaling, see also [22,23].

Moreover, outstandingly, it has been found [30] that the eigenfunction properties of both the 1D Anderson model and the 1D Lloyd’s model (i.e., our generalized 1D Lloyd’s model with α=1), characterized by the *information length* β (defined in Equation (12) below), are *universal* for the fixed ratio of Equation (6). More specifically, it was numerically shown that the scaling function

(7) $$\beta =\frac{\gamma x}{1+\gamma x}\;,$$

with γ∼1, holds; see also [59].

Thus, below we explore the validity of scaling (7) for the eigenfunctions of the generalized 1D Lloyd’s model. Of particular relevance is the regime 0<α<1 where the coexistence of insulating and ballistic regimes has been reported [27] (evidenced by well defined peaks in P(G) at G=0 and G=1); a signature of the coexistence of localized and extended eigenfunctions. In our study, we use the 1D Anderson model as reference.

Alongside this work we generate the random variables {ϵ} needed to construct the generalized 1D Lloyd’s model by the use of the algorithm introduced in Ref. [60]. That algorithm was designed to produce random variables having the probability density function

(8) $$P\left(\epsilon \right)=\frac{C}{\epsilon^{1+\alpha }}\;,$$

which has a lower cut-off at $\epsilon_{0}={\left(C/\alpha \right)}^{1/\alpha }$ such that $\int_{\epsilon_{0}}^{\infty }P\left(\epsilon \right)d\epsilon =1$. In addition, we randomize the signs of the obtained variables {ϵ} in order to set the band center of the generalized 1D Lloyd’s model to E=0. Moreover, we have verified that our conclusions do not depend on our choice of P(ϵ). Indeed, we obtained similar results (not shown here) with $P\left(\epsilon \right)=\alpha /{\left(1+\epsilon \right)}^{1+\alpha }$ and $P\left(\epsilon \right)=\left[1/\Gamma \left(\alpha \right)\right]2^{-\alpha }\exp\left(-1/2\epsilon \right)/\epsilon^{1+\alpha }$ (where Γ is the Euler gamma function), that we also used in [27].

## 2. Results

### 2.1. Extraction of the Localization Length ξ

We obtain the localization length ξ for the generalized 1D Lloyd’s model as follows: We open the isolated wires by attaching two semi-infinite single channel leads to the border sites at opposite sides. Each lead is described by a 1D semi-infinite tight-binding Hamiltonian. Using standard methods, see e.g., [61,62,63,64], we can write the transmission amplitude through the disordered wires as

(9) $$t=-2i\sin\left(k\right)\;W^{\;T}\frac{1}{E-H_{\mathrm{eff}}}W\;,$$

where k=arccos(E/2) is the wave vector supported in the leads and $H_{\mathrm{eff}}$ is an effective non-hermitian Hamiltonian given by $H_{\mathrm{eff}}=H-e^{ik}WW^{\;T}$. Here, W is a L×1 vector that specifies the positions of the attached leads to the wire. In our setup, all elements of W are equal to zero except $W_{11}$ and $W_{L1}$ which we set to unity (i.e., the leads are attached to the wire with a strength equal to the inter-site hopping amplitudes: ν=1). Also, we have fixed the energy at E=0. Therefore, we use Equation (4) to compute *G*.

**Figure 1:**
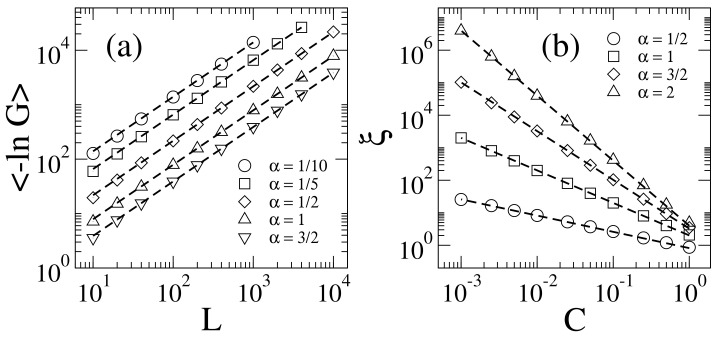
(**a**) Average logarithm of the conductance $\langle -\ln G\rangle$ as a function of *L* for the generalized 1D Lloyd’s model characterized by the values of α indicated on the figure (symbols). C=1 and E=0 were used. Each point was calculated using $10^{4}$ disorder realizations. Dashed lines are the fittings of the data with Equation (5). The obtained values of ξ from the fittings are: ξ≈0.143 (for α=1/10), ξ≈0.304 (for α=1/5), ξ≈0.919 (for α=1/2), ξ≈2.535 (for α=1), and ξ≈5.144 (for α=3/2). (**b**) ξ as a function of the constant C. Dashed lines are the fittings of the data with Equation (10). The obtained values for the proportionality constant *c* from the fittings are: c≈0.82 (for α=1/2), c≈2.01 (for α=1), c≈3.25 (for α=3/2), and c≈4.01 (for α=2). Error bars on both panels are not shown since they are much smaller than symbol size.

Then, in Figure 1a we present the ensemble average $\langle -\ln G\rangle$ as a function of *L* for the generalized 1D Lloyd’s model for several values of α. It is clear from this figure that $\langle -\ln G\rangle \propto L$ for all the values of α we consider here. Therefore, we can extract the localization length ξ by fitting the curves $\langle -\ln G\rangle$ vs. *L* with Equation (5); see dashed lines in Figure 1a. We have observed that it is possible to tune ξ for the same value of α by moving C in Equation (8). Thus, in Figure 1b we report the values of ξ, extracted from the curves $\langle -\ln G\rangle$ vs. *L*, as a function of C. Moreover, note that

(10) $$\xi \propto C^{-\alpha }\;,$$

as shown with the dashed lines in Figure 1b.

In the following Subsection, we use the values of ξ obtained here to construct the ratio of Equation (6) and verify scaling (7) accordingly.

### 2.2. Calculation of the Information Length β

We compute the information length β for the generalized 1D Lloyd’s model by the use of the Shannon entropy *S* of the corresponding eigenfunctions: With the Shannon entropy for the eigenfunction $\Psi^{m}$, which is given as

(11) $$S=-\sum_{n=1}^{L}{\left(\Psi_{n}^{m}\right)}^{2}\ln{\left(\Psi_{n}^{m}\right)}^{2}\;,$$

we write β (see e.g., [65]) as

(12) $$\beta =\exp\left[-\left(S_{\mathrm{GOE}}-\langle S\rangle \right)\right]\;.$$

Here $S_{\mathrm{GOE}}\approx \ln\left(L/2.07\right)$ is the entropy of a random eigenfunction with Gaussian distributed amplitudes (i.e., an eigenfunction of the Gaussian Orthogonal Ensemble [66]). With this definition for *S* (recall that *S* provides the number of principal components of an eigenfunction in a given basis) and β, when the eigenfunctions are localized $\langle S\rangle \approx 0$ and β≈2.07/L, which tends to zero for large *L*. On the other hand, when *fully chaotic* eigenfunctions extend over the *L* available basis states $\langle S\rangle =S_{\mathrm{GOE}}$ and β=1. Therefore, β can take values in the range (0,1].

Below we use exact numerical diagonalization to obtain the eigenfunctions $\Psi^{m}$ (m=1…L) of large ensembles of the generalized 1D Lloyd’s model characterized by the parameters *L*, C, and α. We perform the average $\langle S\rangle$ taking half of the eigenfunctions, around the band center, for each disordered wire length such that $\langle S\rangle$ is computed with $10^{5}$ data values. For example, since for a wire of length $L=10^{3}$ we extract $10^{3}/2$ Shannon entropies only, we construct an ensemble of $2\times 10^{2}$ disordered wires of that length to compute $\langle S\rangle$ from a double average; that is, we average over a subset of the eigenfunctions and over wire realizations. We use half of the eigenfunctions around the band center to be consistent with the fact that we have fixed E=0 in Equation (9), however we have verified that our conclusions do not depend on this choice: i.e., we could reduce the energy window around E=0 or even consider all eigenfunctions to compute $\langle S\rangle$ obtaining equivalent results.

**Figure 2:**
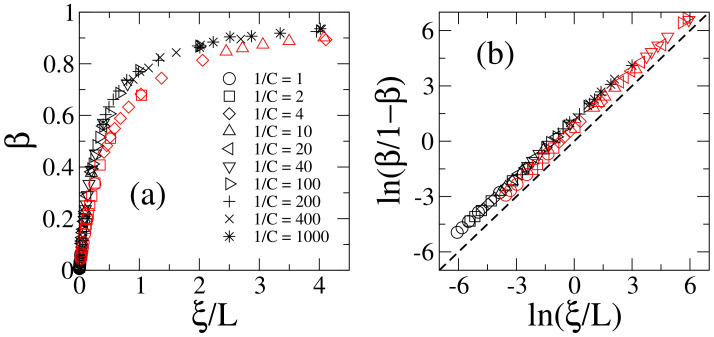
(**a**) Information length β as a function of ξ/L [see Equations (6) and (7)] for the generalized 1D Lloyd’s model with α=1 (black symbols) and the 1D Anderson model (red symbols). (**b**) Logarithm of β/(1−β) as a function of ln(ξ/L) [see Equation (13)]. Different symbols correspond to disorder distributions with different constants C, as indicated in (**a**). Each symbol is computed by averaging over $10^{5}$ eigenstates. Wires with $10^{2}\leq L\leq 10^{3}$ were used. The identity, black dashed line in (**b**), is shown to guide the eye.

In Figure 2a we present β as a function of ξ/L, see Equations (6) and (7), for the generalized 1D Lloyd’s model with α=1, i.e., for the actual 1D Lloyd’s model. Recall that to construct the ratios ξ/L we are using the values of ξ obtained in the previous Subsection from the scaling of Landauer’s conductance; see Figure 1a. In addition, in Figure 2b the logarithm of β/(1−β) as a function of ln(ξ/L) is presented. The quantity β/(1−β) is useful in the study of the scaling properties of β because

(13) $$\frac{\beta }{1-\beta }=\gamma x\;,$$

which is obtained directly by equating γx from (7), implies that a plot of ln[β/(1−β)] vs. ln(x) (with x=ξ/L) is a straight line with unit slope. This fact applies for 1D Lloyd’s model as can be easily verified by comparing the black symbols with the black dashed line in Figure 2b. Note that to construct Figure 2 we have used wires with lengths in the range $10^{2}\leq L\leq 10^{3}$ only, however by changing C (different symbols indicate different values of C) it is possible to span a large range of ξ/L values.

In Figure 2 we include, in red symbols, the corresponding data for the 1D Anderson model. Note that the functional forms in both panels, for both data sets, are very similar. With this, we confirm that scaling (7) is valid for the 1D Lloyd’s model and the 1D Anderson model; as already shown in [30].

**Figure 3:**
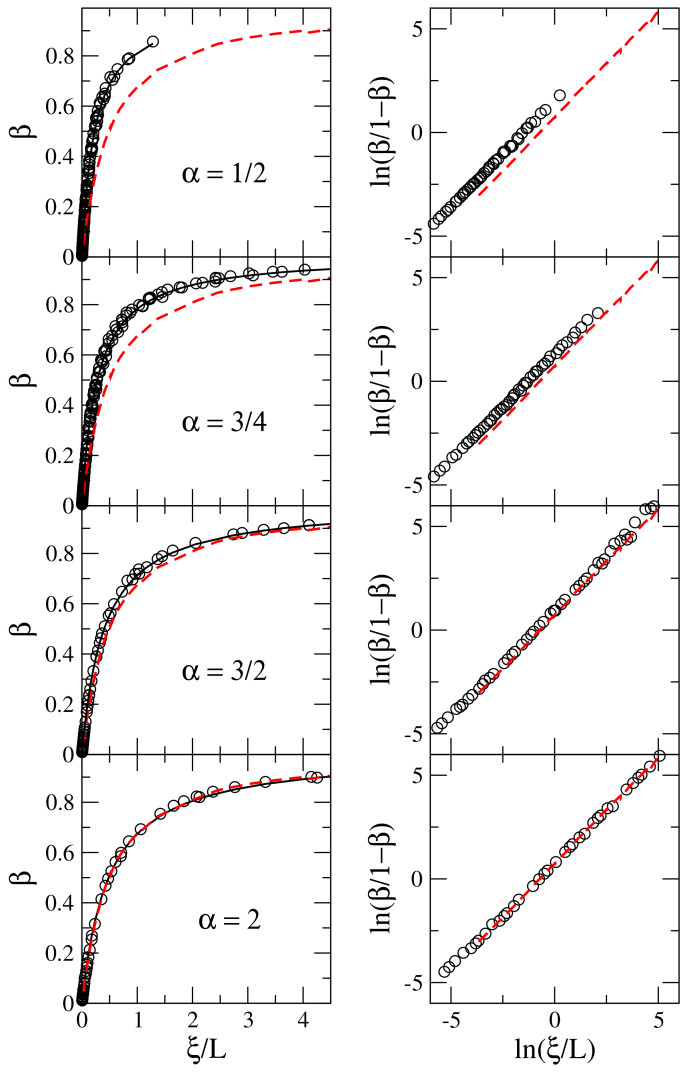
(**Left panels**) Information length β as a function of ξ/L [see Equations (6) and (7)] for ensembles of 1D disordered wires. (**Right panels**) Logarithm of β/(1−β) as a function of ln(ξ/L) [see Equation (13)]. Black circles correspond to the generalized 1D Lloyd’s model with α=1/2, 3/4, 3/2, and 2 (from top to bottom); while the Anderson model is represented by red dashed lines. Each symbol is computed by averaging over $10^{5}$ ($10^{6}$) eigenstates when $L\geq 10^{2}$ ($L<10^{2}$). Here, as in Figure 2, we change C to span a large range of ξ/L values however we do not use different symbols to indicate different values of C to avoid figure saturation. Full black lines in left panels correspond to fittings of the data with Equation (7) with γ≈4.3798 (for α=1/2), γ≈3.5453 (for α=3/4), γ≈2.4891 (for α=3/2), and γ≈2.069 (for α=2).

Now, in Figure 3 we plot curves of β vs. ξ/L (left panels) and ln[β/(1−β)] vs. ln(ξ/L) (right panels) for several values of α. Again, as reference, we include curves for the 1D Anderson model (red dashed lines). In particular, we present representative values of α in the interval 0<α<1 (α=1/2 and 3/4), one value of α in the interval 1≤α<2 (α=3/2), and α=2. We stress the choice of the values of α in Figure 3 because it is known that the properties of systems with Levy-type disorder can be significantly different when the disorder is characterized by values of α in the intervals 0<α<1 or 1≤α<2. However, as shown in Figure 3 there is no major difference between different values of α except for the evolution of the curves towards that for the 1D Anderson model when increasing α. In fact, once α=2 the 1D Anderson model is effectively reproduced, see the lower panels of Figure 3.

**Figure 4:**
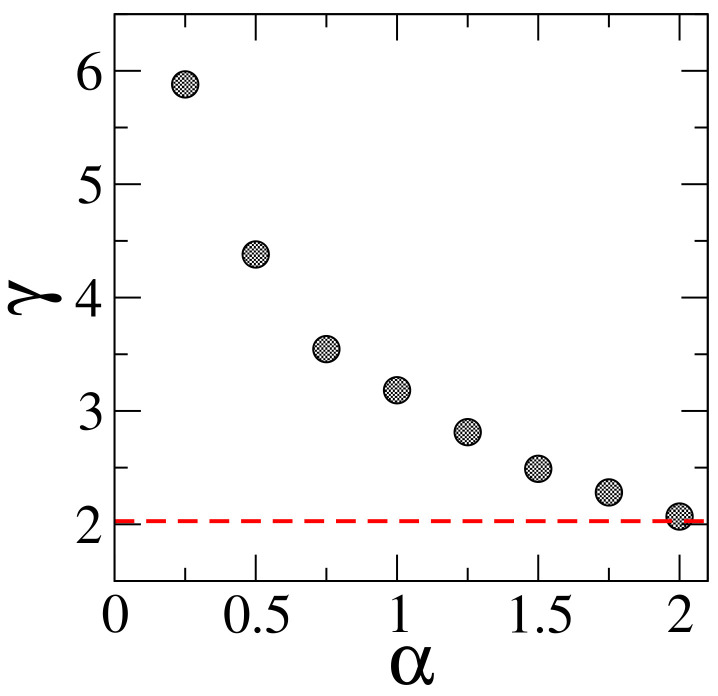
Coefficient γ as a function of α, obtained from the fittings of the curves β vs. ξ/L with Equation (7); see left panels in Figure 3. Black circles correspond to the generalized 1D Lloyd’s model, while the 1D Anderson model is represented by the red dashed line at γ≈2. Error bars are not shown since they are much smaller than symbol size.

Thus, we have verified that scaling (7) describes remarkably well all data sets. Indeed, in left panels of Figure 3 we show fittings of the data with Equation (7); see full black lines. Finally, in Figure 4 we report the values of the coefficients γ, obtained from the fittings of the data using Equation (7), for the values of α considered here. Clearly, when α=2, the coefficient γ is already very close to that for the 1D Anderson model (γ≈2); while, in the opposite limit (i.e., when α→0) the value of γ becomes relatively large.

## 3. Discussion

In this paper, by the use of extensive numerical simulations, we demonstrate that the information length β of the eigenfunctions of our generalization of 1D Lloyd’s model scales with the ratio x=ξ(α)/L as γx/(1+γx), where ξ(α) is the eigenfunction localization length, γ≡γ(α), and *L* is the wire length. Here α is the power-law decay of the long-tailed distributions, $P\left(\epsilon \right)∼1/\epsilon^{1+\alpha }$, characterizing the on-site random energies of 1D tight-binding wires.

It is particularly relevant that scaling (7) describes the eigenfunction properties of our generalization of 1D Lloyd’s model for all values of α, since it has been shown that transport properties of this model are significatively different in the intervals 0<α<1 and 1≤α<2 [27]. Moreover, we have shown that for α=2 the generalized 1D Lloyd’s model already reproduces the properties of the 1D Anderson model.

It is pertinent to add that scaling (7) is also valid for the eigenfunctions of other disordered models (when the scaling parameter *x* is properly defined): the banded random matrix (BRM) model [67,68,69,70,71,72,73,74], the kicked-rotator model [65,71,75] (a quantum-chaotic system characterized by a random-like banded Hamiltonian matrix), the diluted BRM model [76], and multiplex and multilayer random networks [77]. Thus, we include our generalization of 1D Lloyd’s model to the family of complex systems described by scaling (7).

Finally, we want to recall that scaling (7), can be rewritten in a "model independent" form as a relation between properly-defined inverse lengths [30,67]:

(14) $$\frac{1}{d(L,\xi )}=\frac{1}{d(\infty ,\xi )}+\frac{1}{d(L,0)}\;,$$

were $d\left(L,\xi \right)\equiv \exp\left[\langle S(L,\xi )\rangle \right]$, which is also applicable to our generalization of 1D Lloyd’s model.
